# Relationship Between Weight Status and Self-Image Mediated by Pubertal Timing and Athletic Competence: A Cohort Study With Taiwanese Adolescents

**DOI:** 10.3389/fpubh.2022.890751

**Published:** 2022-07-22

**Authors:** Jen-Hao Kuo, Josue Jaru Ubeda Herrera, Chia-Yi Liu, Ting-Hsuan Lee, Carol Strong, Chung-Ying Lin, Yun-Hsuan Chang, Yi-Ching Lin, Yi-Ping Hsieh, Meng-Che Tsai

**Affiliations:** ^1^School of Medicine, College of Medicine, National Cheng Kung University, Tainan, Taiwan; ^2^Department of Business Administration, National Cheng Kung University, Tainan, Taiwan; ^3^Department of Pediatrics, National Cheng Kung University Hospital, College of Medicine, National Cheng Kung University, Tainan, Taiwan; ^4^Department of Public Health, National Cheng Kung University Hospital, College of Medicine, National Cheng Kung University, Tainan, Taiwan; ^5^Institute of Allied Health Sciences, College of Medicine, National Cheng Kung University, Tainan, Taiwan; ^6^Institute of Genomics and Bioinformatics, College of Life Sciences, National Chung Hsin University, Taichung, Taiwan; ^7^Health and Counseling Center, National Chung Hsing University, Taichung, Taiwan; ^8^Institute of Gerontology, National Cheng Kung University, Tainan, Taiwan; ^9^Institute of Behavioral Medicine, College of Medicine, National Cheng Kung University, Taiwan, Taiwan; ^10^Department of Early Childhood and Family Education, College of Education, National Taipei University of Education, Taipei, Taiwan; ^11^Department of Social Work, College of Nursing and Professional Disciplines, University of North Dakota, Grand Forks, ND, United States; ^12^Department of Medical Humanities and Social Medicine, College of Medicine, National Cheng Kung University, Tainan, Taiwan

**Keywords:** self-image, body image, weight status, pubertal timing, athletic performance

## Abstract

**Background:**

Self-image reflects overall self-acceptance in developing adolescents. Using a representative cohort of Taiwanese youth, this study aims to explore the relationship between weight status, pubertal timing, athletic competence, and adolescent self-image.

**Methods:**

Data come from the Taiwan Youth Project that comprised a longitudinal cohort of adolescents (*N* = 2690, 51% males, M*age* = 13.3 ± 0.5 years) surveyed annually from seventh grade. Self-image was measured by perceived satisfaction with appearance and physique. Weight status was proxied by self-reported body mass index (BMI; kg/m^2^). Pubertal timing was defined using the Pubertal Developmental Scale, which mainly measured physical changes in puberty. Athletic competence was assessed by experiences with participation in competitive sports and self-perceived talent for sports. Linear regression analysis was applied to test for an association between BMI and self-image. In order to test for mediating roles of pubertal timing and athletic competence, Hayes' PROCESS macro on SPSS was conducted applying 5,000 bootstrap resamples with 95% confidence intervals of the indirect effect.

**Results:**

BMI was inversely associated with self-image in both males (β = −0.074, [−0.095, −0.053]) and females (β = −0.095, [−0.122, −0.069]). The boot-strapped 95% confidence intervals indicated statistically significant mediating effects of pubertal timing (β = −0.008, [−0.015, −0.001]) and athletic competence (β = −0.006, [−0.011, −0.002]) in the link between BMI and self–image in females, whilst only athletic competence mediated this association (β = −0.006, [−0.009, −0.002]) in males. Moreover, BMI at baseline were also associated with long-term self-image in males (β = −0.037, [−0.057, −0.017]) and females (β = 0.132, [0.073, 0.190]).

**Conclusions:**

Understanding the mediating factors may help enhance adolescents' self-image by providing guidance on healthy weight and physical activity behaviors according to different stages of pubertal progression.

## Introduction

Self-image lies on a continuum from a favorable and positive subjective perception to an unfavorable and negative subjective perception of oneself, which comes from a phenomenological organization of individual experiences and ideas in all aspects of self-influence (1). Self-image is practically determined by one's perceived appearance, physique, and the importance of the appearance and physique associated with one's performance within social groups (2). Particularly during adolescence, physical appearance is crucial to the perceptual self-image (3), and adolescents experience the process of transformation of old self-images with new ones, which involves the adaptability of the individual reorganization (4). Therefore, a positive self-image is correlated with more adaptive adolescents who are easier to internalize the new personal construct, while a negative self-image is in connection with less adaptive adolescents who face the difficulties of restructuring, resulting in the risk of personal dissonance and intrapersonal conflicts (5). More specifically, self-image is considered a vital driver of prosocial behavior and an essential indicator of health status (6), because of its relevance to depressive symptoms (7), suicidal ideation (8), eating disorders (9), and substance use disorders (10).

Puberty is usually marked by sexual maturation and physical growth that may have relevance to psychosocial adaptations during adolescence. Previous studies have shown early puberty to be associated with negative impacts on self-image in both males and females (11–13). Especially in female adolescents, poor self-image is potentially related to external manifestations resulting from early sexual maturation (14). They may be concerned about their physical selves due to cultural expectations for physical attractiveness at the age of initiating dating (15). Besides the cultural perspective on perceptual body image, pubertal hormones interact with the neurocognitive changes related to the social emotion region of the brain (16). Therefore, once the biological changes initiated by pubertal hormones are perceived, adolescents may adjust their body shape to keep up with social standards of high muscle mass and slenderness for boys and girls, respectively (17). That being said from a biobehavioral perspective, the pursuance of an ideal body shape may diverge because of different hormonal actions in females and males, as boys might work out for an ideal muscle and girls might chase or keep up with a slender body shape under social pressures (18). In addition, weight status proxied by the body mass index (BMI) has also been proven to be associated with self-image (19). An increase in BMI typically predicts dissatisfaction with one's body, and this correlation may become more manifested as children become adolescents during pubertal growth (20). Although the impacts of pubertal timing and weight status on adolescent self-image are usually discussed independently, these two predictors are physiologically intertwined potentially due to the interaction between the adipokines (e.g., leptin) and the kisspeptin systems (21). Particular pubescent transformations, including early pubertal timing and high BMI, have been found to be correlated in growing adolescents and result in worse self-reported health (22). Given a well-established link between obesity and early pubertal timing, whether this link suggests the mediating role of pubertal timing in the association between weight status and self-image may require more research to prove.

Moreover, athletic competence is another salient factor when adolescent self-image is concerned. Pubertal development and weight status at the same time have strong implications on athletic competence during adolescence (23). It has been observed that variations in maturity influence athletic competence (24). Youths who are experts in sports tend to have more rapid and advanced sexual maturation as compared with the general population (25). Gender differences have also been noted in this relationship in that early-maturing boys tend to have higher muscular strength than late-maturing boys at all ages between 11 and 17 years, while early-maturing girls may perform only slightly better than late maturing girls in early adolescence (26). Along with weight status, athletic activity also plays a critical role in adolescents' physical and psychological health (27). Regardless of sex or age, some studies suggest that those who participate in sports acquire positive benefits in self-image (28). Sports participation, which may be beneficial for neurogenesis and neurotrophin in brain plasticity correlated with self-efficacy (29), can lead to more positive attitudes about one's own body and self-image (30). After searching for previous similar studies, it was noted that there is no prior research investigating the association between weight status, pubertal timing, athletic competence, and adolescent self-image, and therefore more research is needed to fill this gap in the literature.

### The Present Study

In Taiwan, only a few cross-sectional studies have indicated negative effects of overweight status on self-image (31, 32). However, these studies failed to describe the potential mediation mechanism linking weight status and self-image, except for one study that mainly focused on the mediating role of social class background (33). Therefore, this study is aimed toward examining whether there is a significant association between weight status and adolescent self-image and if so, whether pubertal timing and athletic competence may play mediating roles in this relationship. Further, we tested the longitudinal effects of these salient factors on later self-image using a longitudinal representative youth cohort.

## Materials and Methods

### Study Population

The data used in this study were retrieved from the Taiwan Youth Project (TYP), which was launched by the Institute of Sociology, Academia Sinica, Taiwan (34). In brief, two cohorts of 7th- and 9th-grade students were recruited in 2000 and followed up annually. The participants were recruited from northern Taiwan, including Taipei City, New Taipei City (called Taipei County before 2010), and Yilan County, using a multistage-stratified and class-clustered selection procedure with randomly selected schools and classes. Finally, 40 schools (Taipei City: 16 schools; New Taipei City: 15 schools; Yilan County: 9 schools) and 81 classes in each grade were chosen. All participants consented to participate in the TYP. We only included a subset of data (*N* = 2,690) on the same cohort of seventh graders that collected items related to self-image from wave 1 (M*age* = 13.3 ± 0.5 years in 2000) and wave 9 (M*age* = 22.3 ± 0.5 years in 2009) and thus were deemed valid for the analysis. The study was approved by the Institutional Review Board of the National Cheng Kung University Hospital.

### Measures

#### Weight Status

The body mass index (BMI) was calculated based on self-reported body heights and weights. BMI is a value of a person's leanness or corpulence derived from their height and weight, and it has been proven to have high specificity in diagnosing obesity and related comorbidities in children and teenagers over other indicators (35). The formula is BMI = kg/m^2^, where kg is a person's weight in kilograms, and m^2^ is their height in meters squared.

#### Pubertal Timing

Pubertal timing was assessed based on the items in the Pubertal Developmental Scale (PDS), which measures self-perceived physical changes, including height spurts, body hair development, skin changes, breast growth/deepening of the voice, and menarche/facial hair development (36, 37). This score was chosen because of its benefit in terms of assessing neuroendocrine changes in this phase of life. This scale does not have illustrations of pubertal stages; it does not mention genitalia, nor does it involve being seen naked or palpated. Thus, this scale is extensively used in the literature because it is less embarrassing for youngsters and is cheaper and easier to administer than Tanner ratings (38). Except for menarche, which was a dichotomous item (“yes” or “no”), all other items were rated using a 4-point Likert scale. Aligned with prior research, we added the PDS scores and standardized them within same-sex and same-age cohorts (in years) to represent pubertal timing among the participants (39) where a higher score represented earlier pubertal timing.

#### Athletic Competence

As for athletic competence, the participants were asked to report on their experiences with participation in competitive sports on behalf of their school before and after entry to junior high school and their self-perceived talent for sports. Perceived talent for sports and athletic competence has been shown to be correlated with actual physical fitness and physical activity participation (40, 41), and thus it was used to represent the level of physical fitness that was not measured in the dataset. Every response to these three questions was a dichotomous item (“yes” or “no”), which in the data analysis is presented as 1 = yes and no = 0, and then added to create a single scale that indicates the degree of athletic competence (range 0–3), where a higher score represents a higher level of athletic competence (Cronbach's alpha = 0.661).

#### Self-Image

Given the rapid biological and cognitive changes occurring during puberty, physical appearance is essential to consider when studying self-image during adolescence (3, 42). Self-image was measured using two items that assessed perceived satisfaction with appearance and physique. Each item was rated on a 4-point Likert scale, ranging from 1 (strongly unsatisfied) to 4 (strongly satisfied), and then added to create a single scale that indicated the degree of self-image, where a higher score represents a higher level of self-image (Spearman-Brown coefficient = 0.796).

#### Covariates

Socioeconomic covariates included parental education and family incomes. The parent with the most education of the two parents was used as the reference for parental education. Monthly family income was subdivided into three groups: “New Taiwanese dollar (NTD) 30,000 or less,” “NTD 30,001–60,000,” and “NTD 60,001” or more.

### Statistical Analysis

**Figure 1:**
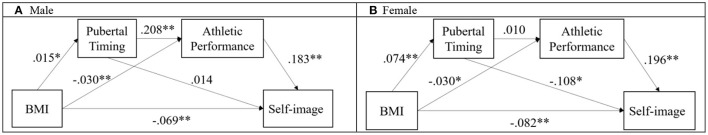
Mediation analysis on pubertal timing and athletic competence in the association between BMI and self-image stratified into male **(A)** and female **(B)** samples. *p < 0.05; **p < 0.01; BMI indicates body mass index.

Using the cross-section data at wave 1, we examined the sequential relationship among BMI, pubertal timing, athletic competence, and self-image. Firstly, linear regression analyses were applied to test for an association between BMI and self-image. Further, to test for an indirect effect of BMI on self-image via potential mediators (i.e., pubertal timing and athletic competence), these variables were included in a series of regression analyses with self-image as the outcome variable. The significance of the mediation effect of BMI and athletic competence on the association between pubertal timing and self-image was examined using the bootstrap method with 5,000 repeated random samples. In total 95% confidence intervals of the indirect effect were calculated, which provides evidence of a statistically significant indirect effect when the interval does not contain zero (43). Specifically, we tested three indirect paths linking pubertal timing to self-image: (1) BMI → pubertal timing → self-image, (2) BMI → athletic competence → self-image, and (3) BMI → pubertal timing → athletic competence → self-image (Figure 1). Given that pubertal timing and BMI were assessed at the same wave, we conducted another mediation analysis on the path from pubertal timing via BMI and athletic performance to self-image, in order to examine the reverse causality between pubertal timing and BMI (see the Supplementary Materials). As to examining the long-term effects of BMI on self-image at wave 9, a full linear regression model included BMI, pubertal timing, athletic competence, and self-image at wave 1 as the independent variables. All the regression and mediation models were analyzed using gender-stratified subsamples and adjusted for parents' education and household income. We conducted statistical analyses using SPSS V.25.0 (SPSS, Chicago, Illinois, USA). The mediation analysis was performed using PROCESS macro 3.5 for SPSS developed by Preacher and Hayes (44).

## Results

### Demographic Characteristics

Of the 2,690 participants, 51.2% were males, and 48.8% were females, with an average age of 13.3 (± 0.5) years at wave 1 (Table 1). The PDS score in females was 11.36 (± 2.10), and in males, it was 9.50 (± 2.27). At wave 1, a slightly higher level of self-image was observed in the males (score = 5.02 ± 1.46) as compared to the females (4.45 ± 1.48), but this difference was not statistically significant (*p* = 0.06). In addition, the majority (*N* = 1,055, 39.2%) of monthly family income ranged between 30,001 and 60,000 NTD, and most of the (*N* = 1,066, 39.6%) parents had attained a senior high school education. Generally, males had higher levels of athletic performance and self-image than their female peers.

**Table 1:** Demographic information of participants (*N* = 2,690).

	**Male (*N =* 1,378)**	**Female (*N =* 1,312)**	***p*-value**
Variables of interest, mean (S.D.)			
Pubertal timing (range 5–20)	9.50 (2.27)	11.36 (2.10)	0.06
Body mass index	20.43 (4.07)	19.58 (3.22)	0.10
Athletic competence (range 0–3)	1.05 (1.08)	0.89 (1.02)	0.03
Self-image, wave 1 (range 1–7)	5.02 (1.46)	4.45 (1.48)	0.04
Self-image, wave 9 (range 1–7)	4.65 (1.14)	4.24 (1.18)	0.03
Covariates, *N* (%)			
Monthly family income (NTD)			0.68
≦30,000	230 (16.7)	213 (16.2)	
30,001–60,000	532 (38.6)	523 (39.9)	
≧60,001	487 (35.3)	453 (34.5)	
Missing	129 (9.4)	123 (9.4)	
Parents' education			0.68
Junior high or lower	443 (32.1)	407 (31.0)	
Senior high	512 (37.2)	554 (42.2)	
College or higher	323 (23.4)	294 (22.4)	
Missing	100 (7.3)	57 (4.3)	

### The Cross-Sectional Effect of BMI on Self-Image and Sequential Mediation Analysis of Pubertal Timing and Athletic Competence at Wave 1

Using a linear regression model (Table 2), we found that BMI was inversely associated with self-image in both males (β = −0.074, [−0.095, −0.053]) and females (β = −0.095, [−0.122, −0.069]). That is, those who had a higher BMI were more likely to report a lower level of self-image. Meanwhile, there was also a positive association between BMI and pubertal timing in both males (β = 0.015, [0.001, 0.030]) and females (β = 0.074, [0.056, 0.092]) and a negative association between BMI and athletic competence in both males (β = −0.030, [−0.045, −0.014]) and females (β = −0.030, [−0.049, −0.011]) (Figure 1). Analyzing the indirect effects with the bootstrapped 95% confidence intervals (Table 2), results revealed that pubertal timing (β = −0.008, [−0.015, −0.001]) and athletic performance (β = −0.006, [−0.011, −0.002]) significantly mediated the relationship between BMI and self-images in females. That is, individuals with a higher BMI value were likely to have earlier pubertal timing and poorer athletic competence which were both associated with poor self-image (Figure 1). Among the males, we only found athletic competence to mediate the association between BMI and self-image (β = −0.005, [−0.009, −0.002]). Nevertheless, the results also suggested that after accounting for the mediating roles, BMI still had a negative impact on self-image (male: β = −0.069, [−0.090, −0.048]; female: β = −0.082, [−0.109, −0.054]).

**Table 2:** Bootstrap test on the effect of pubertal timing and athletic competence on the association between BMI and self-image stratified by gender.

	**Males B (95% CI)**	**Females B (95% CI)**
Total effect	−0.074^**^ (−0.095, −0.053)	−0.095^**^ (−0.122, −0.069)
Direct effect	−0.069^**^ (−0.090, −0.048)	−0.082^**^ (−0.109, −0.054)
Indirect 1	0.000 (−0.001, 0.002)	−0.008^**^ (−0.015, −0.001)
Indirect 2	−0.005^**^ (−0.009, −0.002)	−0.006^**^ (−0.011, −0.002)
Indirect 3	0.001^**^ (0.000, 0.001)	−0.000 (−0.001, 0.001)

* *p < 0.05*;

** *P < 0.01*.

*Indirect 1, BMI → Pubertal timing → Self-image*.

*Indirect 2, BMI → Athletic competence → Self-image*.

*Indirect 3, BMI → Pubertal timing → Athletic competence → Self-image*.

*BMI, body mass index*.

Alternatively, we conducted a mediation analysis on the path from pubertal timing via BMI and athletic performance to self-image, given a potential reverse causality between pubertal timing and BMI (Supplementary Figure S1). We found that pubertal timing was inversely associated with self-image in females (β = 0.173 [−0.261, −0.085]) but not in males (Supplementary Table S1). Meanwhile, the association between pubertal timing and BMI (β = 0.256, [0.009, 0.503]) and athletic competence (β = 0.208, [0.143, 0.273]) was statistically significant in the males, but the association remained significant only between pubertal timing and BMI (β = 0.767, [0.577, 0.957]) in the females. Further, the mediation analysis indicated significant total and indirect (via BMI) effects of pubertal timing on self-image among the female subjects.

### The Longitudinal Association Between Pubertal Timing/ BMI/ Athletic Competence at Wave 1 and Self-Image at Wave 9

Adjusting for self-image at wave 1, we found that pubertal timing was not related to self-image at wave 9 in either males or females (Table 3). In the males, the self-image at wave 9 was related to both BMI (β = −0.037, [−0.057, −0.017]) and athletic competence (β = 0.110, [0.037, 0.182]) at wave 1. In the females, the self-image at wave 9 was only related to BMI at wave 1 (β = 0.132, [0.073, 0.190]). the *p*-value in the males was 0.05 and was 0.001 in the females, supporting the hypothesis tested in this study.

**Table 3:** Regression analysis on the predictors of self-image at wave 9.

	**Males B (95% CI)**	**Females B (95% CI)**
Pubertal timing	−0.050 (−0.131, 0.032)	0.014 (−0.072, 0.100)
BMI	−0.037^**^ (−0.057, −0.017)	−0.079^**^ (−0.106, −0.052)
Athletic competence	0.110^**^ (0.037, 0.182)	0.033 (−0.049, 0.116)
Self-image at wave 1	0.145^**^ (0.090, 0.201)	0.132^**^ (0.073, 0.190)

** *P ≦ 0.01. BMI, Body Mass Index; CI, confidence interval*.

## Discussion

To the best of our knowledge, our study is the first to characterize the mediating role of pubertal timing and athletic competence in the relationship between weight status and self-image using a representative cohort of Taiwanese youth. Overall, we found that a high BMI was linked to negative self-image in both genders. Moreover, athletic competence mediated the abovementioned association in both genders since a linkage between increased BMI levels and decreased athletic performance was found to further lead to low self-image. Meanwhile, pubertal timing was only found to be a significant mediator among the females, which was reflected by earlier pubertal timing that was associated with a high BMI level leading to low self-image. Furthermore, the effect of weight status at baseline on self-image remained significant even in young adults.

Overall, our findings were consistent with prior research in that weight status is considered an important predictor of self-image, whereas our study further found the mediation pathway (45). Diving into the role of pubertal timing in this relationship, the results of this analysis indicated that earlier pubertal timing was associated with increased BMI and poor self-image, where the mediation effect was significant, particularly among the female participants. Correspondingly, prior research has shown that self-image declines in females during early adolescence because of the external manifestations of puberty, such as breast development and menarche (14). In addition, girls may feel devalued when they experience earlier pubertal development and increases in weight (46). This sense of self-devaluation may be aggravated in adolescent girls since they are primarily targeted with stereotypes about their ideal body shape and figure from various sources such as television or the Internet. Neuropsychological research may explain this body-mind linkage because girls with early puberty tend to have higher emotional reactivity, higher heart rate variability, and poorer physical competence, thus leading to poorer self-perception (47, 48). However, a comparative pattern appeared to be insignificant among male adolescents. In a previous study, it was found that males at all age levels tend to relate their body shape, such as the size of their shoulders, to masculinity, and thus they are generally satisfied with body changes inherent in pubertal development (49). In addition, the previous research on this topic reinforces the belief that the social standard of being muscular and slender exists among Taiwanese teenagers and may influence the incidence of body dissatisfaction in this age group (50).

A different covariant that was linked in this study is the mediating role of athletic competence. We found that a higher BMI was associated with poor athletic performance that was further linked to a poor self-image in both genders. Our findings corresponded to the findings of a prior study that showed BMI and leisure-time physical activity were two salient predictive factors of body satisfaction (51). Extending from this common observation, our results indicated that when adolescents develop earlier, they may acquire sports advantages that can be translated into negative or positive assets contributing to the construction of a positive self-image, which may mitigate the negative impacts brought about by BMI scores suggesting they are overweight or obese. However, this relationship was noted only in boys, but not in girls. Physiologically speaking, boys after entering puberty usually gain strength because of higher levels of testosterone, which results in better neuromuscular adaptation (52). The advantage of this change during puberty can be also seen in body changes that enable subjects to enroll in different physical activities. This was supported by a study indicating that early-maturing players have advantageous anthropometrical and physiological development that facilitates domination in sports (53). Another study also showed that biological maturation can be used to predict athletic competence during adolescence because of increased muscular power and high energy that can be applied in sports (54). In terms of biological mechanisms, physical fitness would have blunt effects on hormonal stress-responsive systems, such as the hypothalamic-pituitary-adrenal axis and the sympathetic nervous system, which contribute to reduced emotional and metabolic reactivity as well as increased positive mood and wellbeing. Also, the anti-inflammatory effects of exercise can promote behavioral and metabolic resilience, which exert a positive effect on mental health (55). Further, participation in sports programs may have a positive immediate effect on self-image, notably if the chosen activity is some form of team sport that can give the adolescents some peer support in terms of good opinions about their self-image (56). Therefore, we tentatively conclude that there is a critical mediating role of athletic competence in the relationship between weight status, pubertal timing, and self-image.

In the alternative mediation analysis investigating the path from pubertal timing via BMI and athletic performance to self-image, we only found significant total effects of pubertal timing on self-image in adolescent girls. Meanwhile, a large portion of the impacts were contributed by the indirect link via weight status, highlighting its importance as a determinant of adolescent self-image. Further, in the regression analyses examining the long-term effects on self-image, we found that an earlier level of self-image during adolescence may predict later evaluations of self-image in young adulthood. BMI in both genders and athletic competence in boys were found to be the other significant predictors of the level of a positive self-image in young adulthood, whereas pubertal timing was shown as no longer relevant after a multivariate adjustment. This finding also suggests a persistent effect of weight status on self-image as adolescents grow up. It is worth noting that low self-image may persist if it occurs earlier in adolescence. It is therefore necessary to identify the risks and strengths, such as encouraging participation in sports and a positive attitude toward the weight gain inherent in pubertal development among early-maturing adolescents, in order to build a healthier self-image.

### Implications

Our study identified a potential linkage between weight status and self-image via pubertal timing and athletic competence, where gender differences were clearly noted. The implications of the findings can be 3-fold: Firstly, at the onset of puberty, children should be educated about the potential physical changes they will undergo related to hormonal surges. As the age at pubertal onset has skewed toward being earlier in recent years, and obese children are more likely to be affected (57), such information may need to be provided earlier. Secondly, providing dietary and physical activity guidance or designing age-appropriate nutritional menus and physical education classes may help mitigate the negative impacts brought by being overweight and obese and thus help these individuals build a positive self-image. The school, as the major developmental milieu in which adolescents spend long hours studying and interacting with their peers (58), may also play an active role in reducing the sale of unhealthy food and sugared drinks and promoting physical activities on campus (59). Third, information about physiological body changes should be addressed in the school settings or the media, in order to raise awareness about the importance of accepting oneself and providing ways to increase self-esteem. Last but not least, attention should be given to the influence of exposure to media overemphasizing slimness and fitness. Because body dissatisfaction can be developed in this kind of media environment, adolescents should be provided with education related to media literacy (60). The results highlight the need for parents, teachers, and healthcare providers to assess adolescents' self-image and provide guidance on pubertal progression and physical activity based on different weight statuses.

### Limitations

Our sample has its strength in being a longitudinal cohort study design with a large number of participants and a cluster-based sampling strategy. However, our study had some limitations. First, the study was restricted to a limited choice of questions relevant to self-image. Using two items to evaluate such a complex construct of self-image might be simplistic. Although satisfaction with appearance and physique largely account for self-image during adolescence (61), more questions may be needed to measure the different dimensions of self-image. Secondly, athletic competence was constructed using only three self-reported items rather than standardized fitness tests, such as dashes for speed, vertical and standing long jumps, and distance throwing used to measure coordination and explosive strength (24). These objective measurements may improve or complement the representativeness of physical performance and may require further clarification. Third, the data collected were dated and might not represent the current situation. Readers should be cautious of this time gap between data collection and the proposed secondary data analysis. Despite this, the strength of our study is its longitudinal cohort study design due to collecting prospective observational data over the entire course of the subjects' adolescent period. Analyzing old archived data has arguably been an alternative approach to refining existing literature and inspiring new ideas (62). The analysis should be able to provide some mechanistic explanations regarding the link between weight status and adolescent self-image.

## Conclusion

The results found that adolescents with a higher BMI were more likely to have a lower self-image, and this association could persist into young adulthood. Dissecting sequential mediation mechanisms, we further found that a higher BMI was associated with lower perceived athletic competence and thus further linked to pooer self-image. The finding on the role of pubertal timing was mixed, though. While a higher BMI was linked to an earlier pubertal timing that further led to lower self-image in females, an earlier pubertal timing might contribute to better perceived athletic competence and higher self-image in males. Obtaining knowledge of the mediating factors linking adolescent body and mind may help guide healthy weight and physical activity behaviors according to different stages of pubertal progression.

## Data Availability Statement

Publicly available datasets were analyzed in this study. This data can be found at: The TYP dataset is archived in the Survey Research Data Archive, managed by the Institute of Sociology, Academia Sinica, Taiwan. It requires registration, although it is free and open to the public, when accessing the dataset. All the waves can be found by following this link: https://srda.sinica.edu.tw/browsingbydatatype_result.php?category=surveymethodtype=2csid=1.

## Author Contributions

J-HK and M-CT conceived the study and drafted the manuscript. J-HK, JUH, and C-YL conducted the analysis, while CS, M-CT, and C-YL supervised the analysis. T-HL, Y-CL, Y-HC, and Y-PH contributed the development of study and critically reviewed the manuscript. All authors read and approved the final version of the manuscript.

## Funding

This work was supported by a research grant awarded to M-CT from the Ministry of Science and Technology, Taiwan (108-2629-B-006-002), National Cheng Kung Unviersity Hospital (NCKUH-10602007) and by a Summer Research Project Grant awarded to J-HK from the College of Medicine at National Cheng Kung University (NCKUMCS2019014).

## Conflict of Interest

The authors declare that the research was conducted in the absence of any commercial or financial relationships that could be construed as a potential conflict of interest.

## Publisher's Note

All claims expressed in this article are solely those of the authors and do not necessarily represent those of their affiliated organizations, or those of the publisher, the editors and the reviewers. Any product that may be evaluated in this article, or claim that may be made by its manufacturer, is not guaranteed or endorsed by the publisher.

## References

[1] Bailey JA. Self-image, self-concept, and self-identity revisited. J Natl Med Assoc. (2003) 95:383. 12793794 PMC2594523

[2] Spear BA Barlow SE Ervin C Ludwig DS Saelens BE Schetzina KE. Recommendations for treatment of child and adolescent overweight and obesity. Pediatrics. (2007) 120:S254–88. 10.1542/peds.2007-2329F 18055654

[3] Peng S. Self-image and psychological well-being among Chinese adolescents. University of Missouri–St. Louis. (2012).

[4] Naccache L El Karoui I Salti M Chammat M Maillet M Allali S. Comment notre cohérence subjective se construit-elle? Le modèle de la dissonance cognitive. Bull l'Académie Nat Médecine. (2015) 199:253–9. 10.1016/S0001-4079(19)30968-9 27476307

[5] Harmon-Jones E Harmon-Jones C. Cognitive dissonance processes serve an action-oriented adaptive function. Behav Brain Sci. (2020) 43:e38. 10.1017/S0140525X19002176 32292158

[6] Jordan J Leliveld MC Tenbrunsel AE. The moral self-image scale: Measuring and understanding the malleability of the moral self. Front Psychol. (2015) 6:1878. 10.3389/fpsyg.2015.01878 26696941 PMC4678225

[7] Di Blasi M Cavani P Pavia L Lo Baido R La Grutta S Schimmenti A. The relationship between self-Image and social anxiety in adolescence. Child Adolesc Ment Health. (2015) 20:74–80. 10.1111/camh.12071 32680392

[8] Laukkanen E Honkalampi K Hintikka J Hintikka U Lehtonen J. Suicidal ideation among help-seeking adolescents: association with a negative self-image. Arch Suicide Res. (2005) 9:45–55. 10.1080/13811110590512930 16040579

[9] Erkolahti RK Saarijärvi S Ilonen T Hagman H. Self-image of anorexic and bulimic female adolescents. Nord J Psychiatry. (2002) 56:447–50. 10.1080/08039480260389370 12495540

[10] Naumovska A Bonevski D. Self image and frequent alcohol use in middle adolescence. J US-China Med Sci. (2012) 9:38–44. 10.17265/1548-6648/2012.01.005

[11] Nacinovich R Buzi F Oggiano S Rossi S Spada S Broggi F. Body experiences and psychopathology in idiopathic central precocious and early puberty. Minerva Pediatr. (2016) 68:11–8. 26864719

[12] Mercader-Yus E Neipp-López MC Gómez-Méndez P Vargas-Torcal F Gelves-Ospina M Puerta-Morales L. Anxiety, self-esteem and body image in girls with precocious puberty. Rev Colomb Psiquiatr. (2018) 47:229–36. 10.1016/j.rcpeng.2017.05.015 30286845

[13] Lee JE Ahn HY Choi HS. A study of body image, self-esteem and depression in girls with precocious puberty and normal girls. Adv Sci Techno Lett. (2015) 116:21–5.

[14] Slap GB Khalid N Paikoff RL Brooks-Gunn J Warren MP. Evolving self-image, pubertal manifestations, and pubertal hormones: Preliminary findings in young adolescent girls. J Adolescent Health. (1994) 15:327–35. 10.1016/1054-139X(94)90606-8 7918506

[15] Pritchard ME King SL Czajka-Narins DM. Adolescent body mass indices and self-perception. Adolescence. (1997) 32:863–72. 9426809

[16] Goddings AL Burnett Heyes S Bird G Viner RM Blakemore SJ. The relationship between puberty and social emotion processing. Dev Sci. (2012) 15:801–11. 10.1111/j.1467-7687.2012.01174.x 23106734 PMC3795450

[17] Headley S. A longitudinal study of pubertal timing and extreme body change behaviors among adolescent boys and girls. Youth Studies Australia. (2005) 24:62–3. 15230071

[18] Pletsch K Johnson MK Tosi CB Thurston CA Riesch SK. Self-image among early adolescents: revisited. J Commun Health Nurs. (1991) 8:215–31. 10.1207/s15327655jchn0804_4 1744667

[19] Mäkinen M Marttunen M Komulainen E Terevnikov V Puukko-Viertomies LR Aalberg V. Development of self-image and its components during a one-year follow-up in non-referred adolescents with excess and normal weight. Child Adolesc Psychiatry Ment Health. (2015) 9:1–9. 10.1186/s13034-015-0038-7 25737741 PMC4347918

[20] Coelho EM Padez C Moreira P Rosado V Mourão-Carvalhal I. BMI and self-perceived body shape in Portuguese children. Rev Psicol Deporte. (2013) 22:371–6.

[21] Reinehr T Roth CL. Is there a causal relationship between obesity and puberty? Lancet Child Adolescent Health. (2019) 3:44–54. 10.1016/S2352-4642(18)30306-7 30446301

[22] Hoyt LT Niu L Pachucki MC Chaku N. Timing of puberty in boys and girls: implications for population health. SSM-population Health. (2020) 10:100549. 10.1016/j.ssmph.2020.100549 32099893 PMC7030995

[23] Anderson CB Mâsse LC Zhang H Coleman KJ Chang S. Ethnic, gender, and BMI differences in athletic identity in children and adolescents. J Phys Activity Health. (2011) 8:200–9. 10.1123/jpah.8.2.200 21415447

[24] Beunen G Malina RM. Growth and biologic maturation: relevance to athletic performance. The young athlete. (2008) 1:3–17. 10.1002/9780470696255.ch1

[25] Bastos F Hegg R. The relationship of chronological age, body build, and sexual maturation to handgrip strength in schoolboys ages 10 to 17 years. In: JAP Perspectives in Kinanthropometry. Champaign: Human Kinetics. (1986) p. 45–9.

[26] Malina RM. Secular trends in growth, maturation and physical performance: a review. Anthropol Rev. (2004) 67:3–31. 10.5040/9781492596837.ch-029

[27] Neumark-Sztainer D Paxton SJ Hannan J Haines J Story M Does body satisfaction matter? Five-year longitudinal associations between body satisfaction and health behaviors in adolescent females and males. J Adolescent Health. (2006) 39:244–51. 10.1016/j.jadohealth.2005.12.001 16857537

[28] Markland D Ingledew DK. The relationships between body mass and body image and relative autonomy for exercise among adolescent males and females. Psychol Sport Exerc. (2007) 8:836–53. 10.1016/j.psychsport.2006.11.002

[29] Voss MW Vivar C Kramer AF van Praag H. Bridging animal and human models of exercise-induced brain plasticity. Trends Cogn Sci. (2013) 17:525–44. 10.1016/j.tics.2013.08.001 24029446 PMC4565723

[30] Eide R The The relationship between body image self-image and physical activity. Scandinavian J Soc Med. (1982) 29:109–112. 6958033

[31] Lee J-I Yen C-F. Associations between body weight and depression, social phobia, insomnia, and self-esteem among Taiwanese adolescents. Kaohsiung J Med Sci. (2014) 30:625–30. 10.1016/j.kjms.2014.09.005 25476101 PMC11916389

[32] Noh JW Kwon YD Yang Y Cheon J Kim J. Relationship between body image and weight status in east Asian countries: comparison between South Korea and Taiwan. BMC Public Health. (2018) 18:1–8. 10.1186/s12889-018-5738-5 29970058 PMC6029392

[33] Chen HC. The self-image of Taiwanese adolescents: Gender and social class comparisons. The University of Arizona. (1993).

[34] Tsai MC Strong C Lin CY. Effects of pubertal timing on deviant behaviors in Taiwan: a longitudinal analysis of 7th-to 12th-grade adolescents. J Adolesc. (2015) 42:87–97. 10.1016/j.adolescence.2015.03.016 25956430

[35] Reilly JJ. Diagnostic accuracy of the BMI for age in paediatrics. Int J Obesity. (2006) 30:595–597. 10.1038/sj.ijo.0803301 16570088

[36] Gau SF Soong WT Tsai WY Chiu Y N A Chinese version of a self-administered rating scale for pubertal development. Taiwanese J Psychiatry. (1997) 11:128–40.

[37] Lee CT Tsai MC Lin CY Strong C. Longitudinal effects of self-report pubertal timing and Menarcheal age on adolescent psychological and behavioral outcomes in female youths from Northern Taiwan. Pediatr Neonatol. (2017) 58:313–20. 10.1016/j.pedneo.2016.04.004 27600751

[38] Pompéia S Zanini GD Freitas RS Inacio LM Silva FC Souza GR. Adapted version of the Pubertal Development Scale for use in Brazil. Rev Saude Publica. (2019) 53:56. 10.11606/s1518-8787.2019053000915 31432913 PMC6703897

[39] Carskadon MA Acebo C. A self-administered rating scale for pubertal development. J Adolescent Health. (1993) 14:190–5. 10.1016/1054-139X(93)90004-9 8323929

[40] Jaakkola T Washington T. Measured and perceived physical fitness, intention, and self-reported physical activity in adolescence. Adv Phys Educ. (2011) 1:16. 10.4236/ape.2011.12004

[41] Plante TG Chizmar L Owen D. The contribution of perceived fitness to physiological and self-reported responses to laboratory stress. Int J Stress Manag. (1999) 6:5–19. 10.1023/A:1021906202898

[42] Choi M-S Kim E-Y. Body image and depression in girls with idiopathic precocious puberty treated with gonadotropin-releasing hormone analogue. Ann Pediatric Endocrinol Metabolism. (2016) 21:155. 10.6065/apem.2016.21.3.155 27777908 PMC5073162

[43] Tsai MC Strong C Chen WT Lee CT Lin CY. Longitudinal impacts of pubertal timing and weight status on adolescent Internet use: Analysis from a cohort study of Taiwanese youths. PLoS ONE. (2018) 13:e0197860. 10.1371/journal.pone.0197860 29795649 PMC5967734

[44] Preacher KJ Hayes AF. SPSS and SAS procedures for estimating indirect effects in simple mediation models. Behav Res Methods, Instr Comput. (2004) 36:717–31. 10.3758/BF03206553 15641418

[45] Alsaker FD. Pubertal timing, overweight, and psychological adjustment. J Early Adolescence. (1992) 12:396–419. 10.1177/0272431692012004004

[46] O'Dea JA Abraham S. Association between self-concept and body weight, gender, and pubertal development among male and female adolescents. Adolescence. (1999) 34:69. 10234368

[47] Wojniusz S Callens N Sütterlin S Andersson S De Schepper J Gies I. Cognitive, emotional, and psychosocial functioning of girls treated with pharmacological puberty blockage for idiopathic central precocious puberty. Front Psychol. (2016) 7:1053. 10.3389/fpsyg.2016.01053 27462292 PMC4940404

[48] Schoelwer MJ Donahue KL Didrick P Eugster EA. One-year follow-up of girls with precocious puberty and their mothers: do psychological assessments change over time or with treatment? Horm Res Paediatr. (2017) 88:347–53. 10.1159/000479688 28926827 PMC5808430

[49] Siegel JM Yancey AK Aneshensel CS Schuler R. Body image, perceived pubertal timing, and adolescent mental health. J Adolescent Health. (1999) 25:155–65. 10.1016/S1054-139X(98)00160-8 10447043

[50] Wright MR. Body image satisfaction in adolescent girls and boys: A longitudinal study. J Youth Adolescence. (1988). 18:71–83. 10.1007/BF02139247 24271605

[51] Chen LJ Fox KR Haase AM Ku W. Correlates of body dissatisfaction among Taiwanese adolescents. Asia Pac J Clin Nutr. (2010) 19:172–9. 10.6133/apjcn.2010.19.2.03 20460229

[52] Almeida-Neto PF de Matos DG Pinto VC Dantas PM Cesário TD da Silva LF., Can the neuromuscular performance of young athletes be influenced by hormone levels and different stages of puberty? Int J Environ Res Public Health. (2020) 17:5637. 10.3390/ijerph17165637 32764284 PMC7460253

[53] Itoh R Hirose N. Relationship among biological maturation, physical characteristics, and motor abilities in youth elite soccer players. J Strength Condition Res. (2020) 34:382–8. 10.1519/JSC.0000000000003346 31469763

[54] Kramer T Huijgen BC Elferink-Gemser MT Visscher C. Prediction of tennis performance in junior elite tennis players. J Sports Sci Med. (2017) 16:14. 28344446 PMC5358024

[55] Silverman MN Deuster PA. Biological mechanisms underlying the role of physical fitness in health and resilience. Interface Focus. (2014) 4:20140040. 10.1098/rsfs.2014.0040 25285199 PMC4142018

[56] Bluechardt MH Wiener J Shephard RJ. Exercise programmes in the treatment of children with learning disabilities. Sports Med. (1995) 19:55–72. 10.2165/00007256-199519010-00005 7740247

[57] Huang JY Li CS Chang HP. The age distribution among children seeking medical treatment for precocious puberty in Taiwan. Int J Environ Res Public Health. (2020) 17:6765. 10.3390/ijerph17186765 32957428 PMC7559721

[58] Tsai MC Chou YY Lin SJ Lin SH. Factors associated with adolescents' perspectives on health needs and preference for health information sources in Taiwan. Arch Dis Child. (2013) 98:9–15. 10.1136/archdischild-2012-301629 22820106

[59] Lowry R Galuska DA Fulton JE Wechsler H Kann L. Weight management goals and practices among US high school students: associations with physical activity, diet, and smoking. J Adolescent Health. (2002) 31:133–44. 10.1016/S1054-139X(01)00408-6 12127383

[60] Swami V Taylor R Carvalho C. Body dissatisfaction assessed by the Photographic Figure Rating Scale is associated with sociocultural, personality, and media influences. Scand J Psychol. (2011) 52:57–63. 10.1111/j.1467-9450.2010.00836.x 20626706

[61] Marcoux D. Cosmetics, skin care, and appearance in teenagers. In: Seminars in Cutaneous Medicine and Surgery. WB Saunders. (1999). 10.1016/S1085-5629(99)80022-4 10468044

[62] Dunn SL Arslanian-Engoren C DeKoekkoek T Jadack R Scott LD. Secondary data analysis as an efficient and effective approach to nursing research. West J Nurs Res. (2015) 37:1295–307. 10.1177/0193945915570042 25656875

