# Supplementary material for: Relationship Between Weight Status and Self-Image Mediated by Pubertal Timing and Athletic Competence: A Cohort Study With Taiwanese Adolescents
Source: Front Public Health. 2022 Jul 22;10:890751. doi: 10.3389/fpubh.2022.890751 (PMC9353071; doi:10.3389/fpubh.2022.890751)
Supplement: Supplementary file 1 [file Data_Sheet_1.pdf]

**Table S1.**

*Bootstrap test on the effect of BMI and athletic competence on the association between pubertal timing and self-image stratified by gender*

| | Males β (95% CI) | Females β (95% CI) |
|---|---|---|
| Total effect | .0333 (-.0545, .1212) | .1729** (-.2605, -.0854) |
| Direct effect | .0142 (-.0727, .1011) | -.1077* (-.1954, -.0199) |
| Indirect 1 | -.0177* (-.0367, -.0004) | -.0627* (-.0896, -.0378) |
| Indirect 2 | .0382* (.0203, .0594) | .0019 (-.0109, .0145) |
| Indirect 3 | -.0014* (-.0033, -.0001) | -.0045* (-.0085, -.0014) |

*p<0.05 **P<0.01

Indirect 1, Pubertal timing → BMI → Self-image

Indirect 2, Pubertal timing → Athletic competence → Self-image

Indirect 3, Pubertal timing → BMI → Athletic competence→ Self-image

BMI indicates body mass index.

**Figure S1.**

*Mediation analysis on BMI and athletic competence in the association between pubertal timing and self-image stratified into male (A) and female (B) samples.*

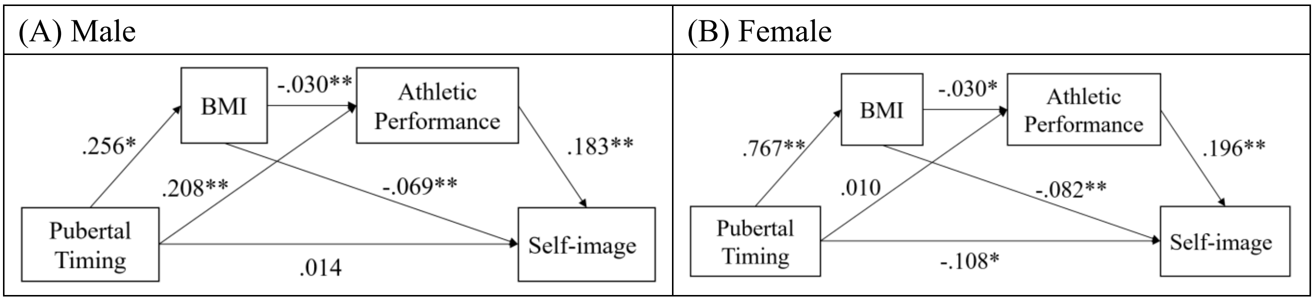


*p<0.05; **p<0.01;

BMI indicates body mass index.
